# Delivery of Platelet-Derived Growth Factor as a Chemotactic Factor for Mesenchymal Stem Cells by Bone-Mimetic Electrospun Scaffolds

**DOI:** 10.1371/journal.pone.0040831

**Published:** 2012-07-12

**Authors:** Matthew C. Phipps, Yuanyuan Xu, Susan L. Bellis

**Affiliations:** 1 Department of Physiology and Biophysics, University of Alabama at Birmingham, Birmingham, Alabama, United States of America; 2 Department of Biomedical Engineering, University of Alabama at Birmingham, Birmingham, Alabama, United States of America; Massachusetts Institute of Technology, United States of America

## Abstract

The recruitment of mesenchymal stem cells (MSCs) is a vital step in the bone healing process, and hence the functionalization of osteogenic biomaterials with chemotactic factors constitutes an important effort in the tissue engineering field. Previously we determined that bone-mimetic electrospun scaffolds composed of polycaprolactone, collagen I and nanohydroxyapatite (PCL/col/HA) supported greater MSC adhesion, proliferation and activation of integrin-related signaling cascades than scaffolds composed of PCL or collagen I alone. In the current study we investigated the capacity of bone-mimetic scaffolds to serve as carriers for delivery of an MSC chemotactic factor. In initial studies, we compared MSC chemotaxis toward a variety of molecules including PDGF-AB, PDGF-BB, BMP2, and a mixture of the chemokines SDF-1α, CXCL16, MIP-1α, MIP-1β, and RANTES. Transwell migration assays indicated that, of these factors, PDGF-BB was the most effective in stimulating MSC migration. We next evaluated the capacity of PCL/col/HA scaffolds, compared with PCL scaffolds, to adsorb and release PDGF-BB. We found that significantly more PDGF- BB was adsorbed to, and subsequently released from, PCL/col/HA scaffolds, with sustained release extending over an 8-week interval. The PDGF-BB released was chemotactically active in transwell migration assays, indicating that bioactivity was not diminished by adsorption to the biomaterial. Complementing these studies, we developed a new type of migration assay in which the PDGF-BB-coated bone-mimetic substrates were placed 1.5 cm away from the cell migration front. These experiments confirmed the ability of PDGF-BB-coated PCL/col/HA scaffolds to induce significant MSC chemotaxis under more stringent conditions than standard types of migration assays. Our collective results substantiate the efficacy of PDGF-BB in stimulating MSC recruitment, and further show that the incorporation of native bone molecules, collagen I and nanoHA, into electrospun scaffolds not only enhances MSC adhesion and proliferation, but also increases the amount of PDGF-BB that can be delivered from scaffolds.

## Introduction

Bone has a dramatic capacity for regeneration following injury, and undergoes constant remodeling during homeostasis. This remarkable regenerative process is initiated by recruitment and differentiation of progenitor cells of mesenchymal origin along with inflammatory cells in order to first form granulation tissue, followed by hyaline cartilage, endochondral ossification and finally the restoration of normal bone structure during remodeling. These activities are coordinated and controlled by an intricate system of growth factors and cytokines/chemokines, such as TGF-β, PDGF, FGF-2, and BMP-2 [1].

Despite bone’s regenerative capability, certain types of bone injuries or pathologies are not able to heal properly, and require intervention in the form of either bone grafts or engineered biomaterials that induce osteoregeneration. Biomaterials designed for bone repair typically serve as a carrier system for delivery of ex vivo-expanded mesenchymal stem cells (MSCs), or alternatively provide a supportive matrix for the attachment and growth of endogenous MSCs that migrate into the implant site. MSCs are multipotent cells within bone marrow (and other tissues) and these cells are a prime candidate for cell-based therapies involving regeneration of bone and other connective tissues [2]. Nonetheless, the inability to efficiently target these cells to selected tissues is a barrier to implementation of MSC therapy [3]. The identification of chemotactic factors for MSCs is crucial in this regard, however there is less known concerning optimal chemoattractants for MSCs when compared with other cell types such as vascular or immune cells.

Platelet-Derived Growth Factor (PDGF) is a polypeptide growth factor that is secreted from cytokine-laden granules of aggregated platelets early after tissue injury [4], [5]. The active form of PDGF, consisting of either a homo- or heterodimer, functions by binding to cell-surface receptors on most cells of mesenchymal origin [6], [7], and participates in the development and remodeling of multiple tissue types, including bone [6]. The potent stimulatory effects of PDGF as a chemoattractant [8], [9] and a mitogen [10], [11], along with its ability to promote angiogenesis [12], [13], position it as a key regulatory molecule in tissue repair. PDGF has been studied in a variety of preclinical models for safety [14], [15] and tissue regeneration as well as clinical trials in periodontal and orthopedic patients [13], [16], [17], [18]. These combined studies have confirmed the effectiveness of PDGF in the repair of musculoskeletal tissue defects. However, the specific molecular mechanisms by which PDGF regulates the activity of multiple cell types to control tissue development require further elucidation. Much of the research in this area has focused on the role of PDGF in controlling vascularization of the nascent tissue forming within the wound site [19].

**Figure 1:**
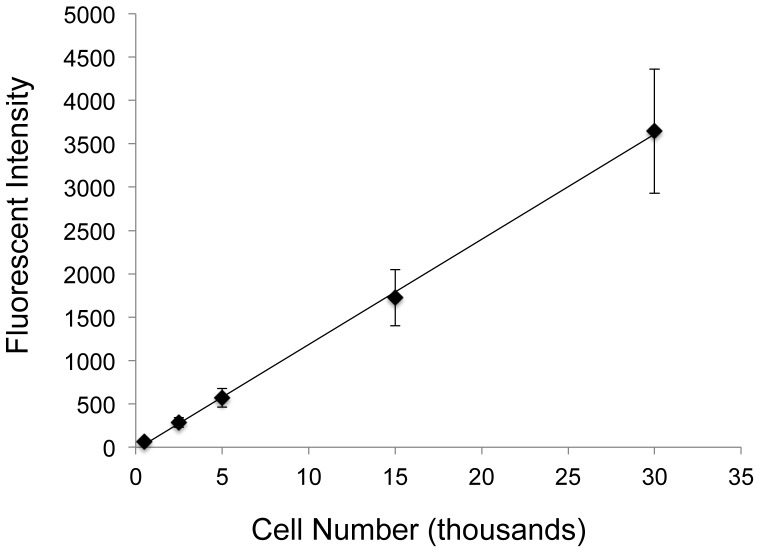
Standard curve of GFP fluorescent signal from lysed GFP-MSCs. GFP-MSCs were counted using a hemocytometer and set numbers of cells were spun down in a centrifuge. Cell pellets were lysed and solution fluorescence was measured by a fluorometer. The coefficient of determination for the linear regression was 0.999, showing a very strong linear correlation between GFP-MSC number and solution fluorescence.

Despite its potency, the half-life of PDGF within blood is only a few minutes [20], indicating that a sustained local delivery of the growth factor will be critical to achieve clinical success. To date, PDGF has been utilized mostly with various carriers in animal models or clinical investigations to overcome the limitation of the short half-life. Examples of previous delivery strategies include: 1) encapsulating PDGF in porous scaffolds or microspheres [21], [22], [23], [24], [25], 2) a heparin-controlled delivery system [26], 3) modification of PDGF with a collagen-binding motif for coupling to collagen carriers [27], and 4) chemical cross-linking of PDGF to demineralized bone matrix [28]. All of these approaches were successful to some degree in extending the PDGF release profile.

In the current study we tested whether delivery of PDGF from bone-mimetic electrospun scaffolds would be effective in stimulating MSC chemotaxis. Electrospinning is a promising and technically-straightforward approach for generating materials that have a porous, nanofibrous architecture similar to native extracellular matrix [29], [30], [31], [32], [33], and this method also allows synthesis of composite substrates incorporating native matrix molecules. Previously we reported that bone-mimetic electrospun scaffolds consisting of blended nanofibers of PCL and collagen I, with embedded nanoparticles of HA, supported greater MSC attachment, survival, proliferation and activation of integrin-related signaling cascades than scaffolds composed of either PCL or collagen I alone [34]. In addition, bone-mimetic scaffolds adsorbed increased amounts of the integrin-binding cell adhesive proteins, fibronectin and vitronectin, from serum, or following implantation into rat tibiae [34]. These results highlighted the benefit of blending the favorable mechanical properties of PCL with the biochemical cues provided by collagen I and nanoHA. We now show that electrospun scaffolds incorporating collagen I and nanoHA, as compared with scaffolds composed of PCL alone, adsorb and release greater quantities of PDGF-BB, leading to enhanced MSC chemotaxis.

**Figure 2:**
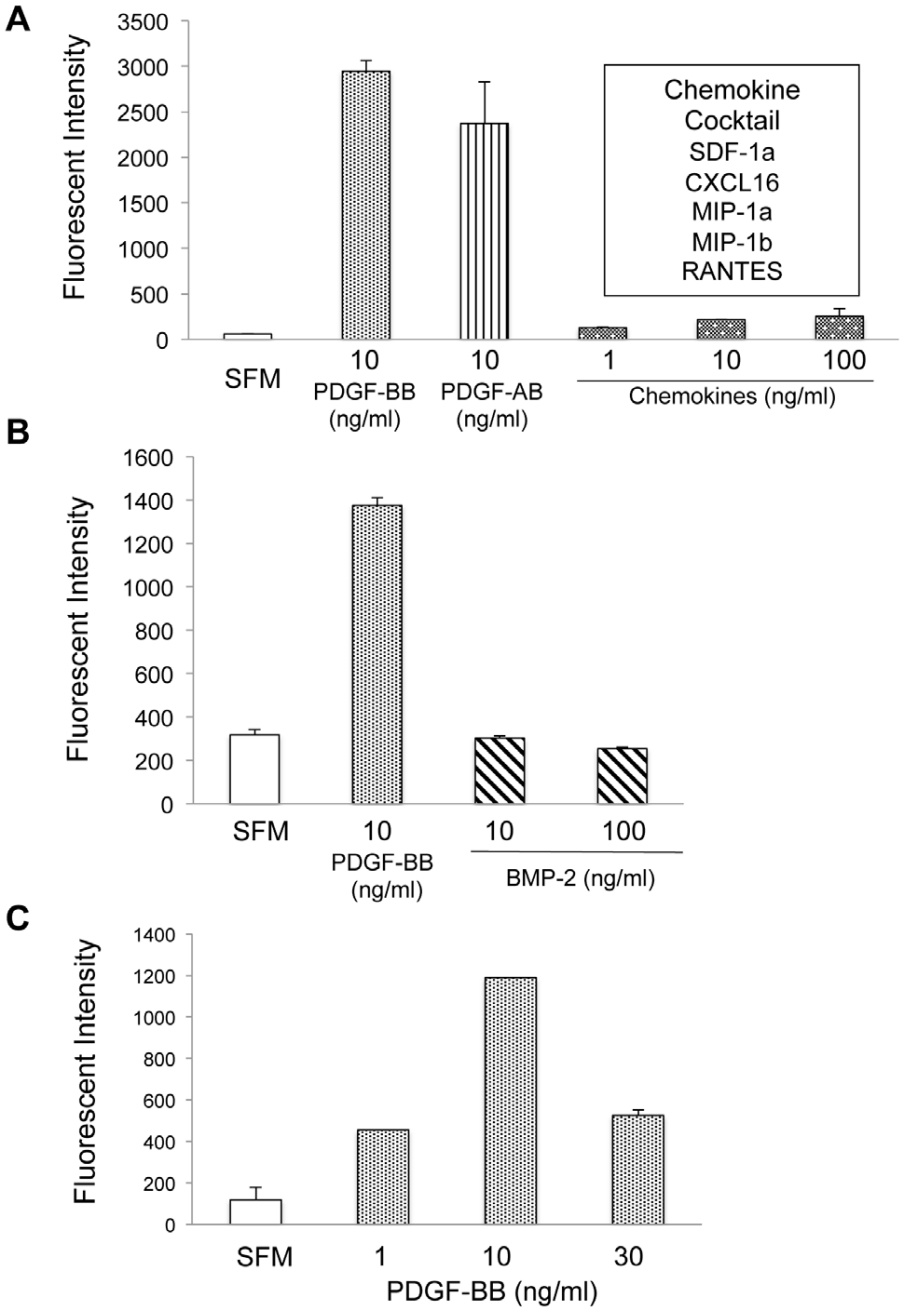
Chemotactic responses of MSCs. GFP-expressing MSCs (4×10^4^) were seeded onto the top of transwell chambers, with various cytokines/chemokines placed in the bottom of the chambers; some wells contained serum-free media (SFM) as a negative control. After a 20 hr incubation at 37°C, the GFP-MSCs that had migrated across the transwell membrane were lysed and quantitated by measuring fluorescence intensity of GFP. The following chemoattractants were evaluated: ***A)*** recombinant human PDGF-BB, PDGF-AB, or a mixture of SDF-1α, CXCL16, MIP-1α, MIP-1β, and RANTES, each at the indicated concentrations (ng/mL) (representative of 3 independent runs) ***B)*** PDGF-BB and BMP-2 (representative of 3 independent runs) and ***C),*** varying concentrations of PDGF-BB showing dose response.

## Materials and Methods

### GFP-MSCs

GFP-expressing human MSCs were obtained from Texas A&M University Health Sciences Center, Institute for Regenerative Medicine. The MSCs were analyzed extensively by the provider institute for cell growth characteristics, osteoblast, adipocyte, and chondrocyte lineage differentiation, and also selected surface markers using flow cytometry. Cells were cultured in αMEM with 2 mM L-Glutamine, and 16.5% Fetal Bovine Serum, as recommended by the provider. Some experiments used reduced serum media, as noted. Cells used in all experiments were passages 2–7.

### PCL/col/HA Scaffold Preparation

PCL/col/HA or 100% PCL scaffolds were prepared as described previously [34], [35]. Briefly, the tri-component scaffolds were electrospun from a 2 mL mixture of polycaprolactone (PCL, MW 100,000), type-I collagen from calf skin (col), and hydroxyapatite (HA) nanoparticles (20–70 nm in size) in a total of 0.262 grams with a dry weight ratio of 50∶30:20, respectively, in hexafluoroisopropanol solvent (HFP) (Sigma-Aldrich, St Louis, MO). The polymer solution was filled in a syringe with a 27-gauge needle, placed in a syringe pump, and the solution was electrospun onto an aluminum foil-grounded target at rate of 2 mL/h under approximately 16–22 KV voltage (Gamma High Voltage Research, Ormond Beach, FL). After electrospinning, residual HFP solvent in the scaffolds was removed by placing scaffolds at room temperature in a vacuumed desiccator for 72 h. PCL, Col, and HA were purchased from Scientific Polymers (Ontario, NY), MP Biomedicals (Solon, OH), and Berkeley Advanced Biomaterials (Berkeley, CA), respectively.

**Figure 3:**
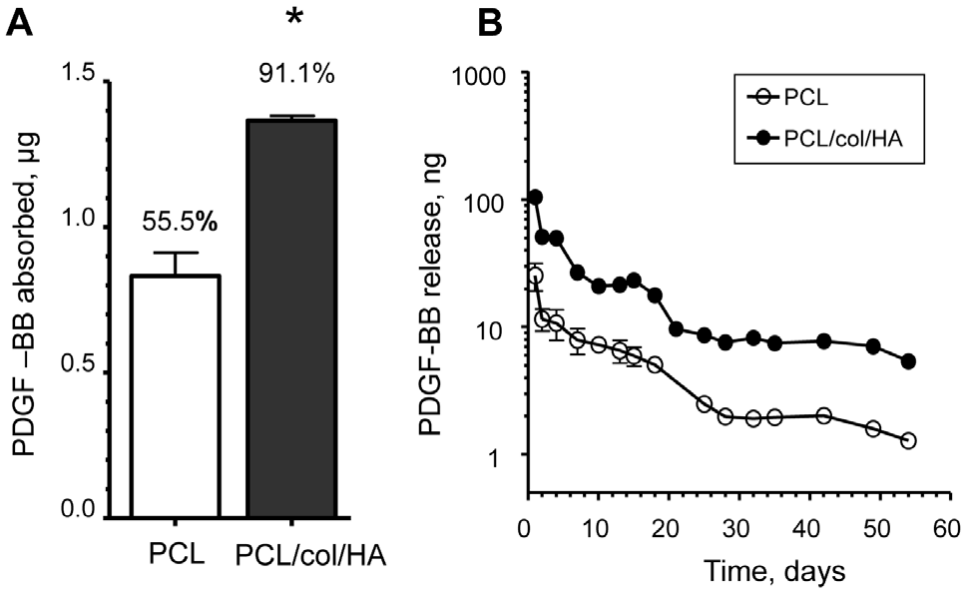
Adsorption and release of PDGF-BB from scaffolds. *A)* Scaffolds were incubated in PBS containing 1.5 µg PDGF-BB for 24 h at 4°C. ELISA assays were used to measure the unbound PDGF-BB in the supernatants. Adsorption of PDGF-BB to the scaffolds was determined by subtracting the unbound PDGF-BB from the 1.5 µg of PDGF-BB initially added. Data are from three independent experiments (* denotes p<0.01). ***B)*** ELISAs were used to measure the amounts of PDGF-BB in conditioned PBS solution collected from the scaffolds at the indicated time intervals over a period of 8 weeks (for many of the data points, error bars are too small to be visualized).

**Figure 4:**
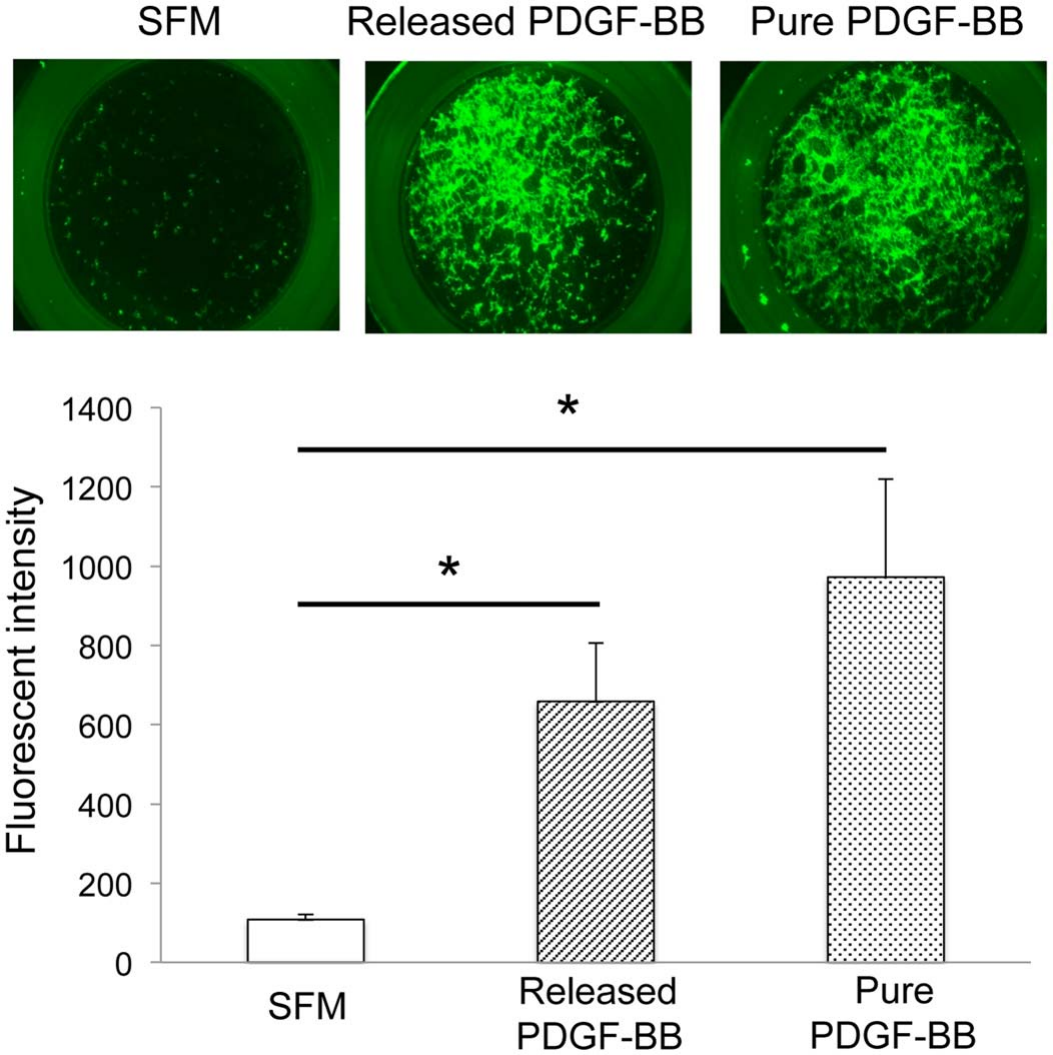
PDGF-BB released from PCL/col/HA scaffolds stimulates MSC chemotaxis. The lower wells of transwell chambers were filled with either purified PDGF-BB (10 ng/mL), serum-free medium (SFM) or PDGF-BB-containing conditioned media collected from PDGF-BB-coated PCL/col/HA scaffolds after 72 hrs. GFP-MSCs were seeded in the upper chambers and allowed to migrate for 20 hrs. After this interval, MSCs adherent to the underside of the transwells were visualized by fluorescent microscopy (top panel, representative images). In addition, MSC migration to the underside of the filter was quantified by lysing cells and measuring solution fluorescence (lower panel). Three independent experiments were performed for solution fluorescence. Analysis of variance with Tukey’s HSD post-hoc was used to establish significance (* denotes *p*<.01).

### Adsorption and Release of PDGF-BB from Scaffolds

Purified recombinant human PDGF-BB (1.5 µg, Leinco Technologies Inc., St. Louis, MO) was passively absorbed to PCL/col/HA or PCL scaffolds (*diameter* = 11 mm, *area* = 95 mm^2^) in 300 µL of PBS at 4°C for 24 h. The amount of PDGF-BB remaining in the solution after incubation with scaffolds (representing the unbound fraction) was measured using an ELISA kit (R&D Systems, Minneapolis, MN). PDGF-BB adsorption by the scaffolds was quantified by subtracting the unbound PDGF-BB from the total amount of protein initially added to the scaffolds (1.5 µg).

To measure release, scaffolds were coated with PDGF-BB as above, rinsed briefly with PBS, and then placed in 5 mL sterile plastic tubes containing 1 mL of PBS, pH 7.2, containing 1% BSA. The scaffolds were incubated at 37°C with gentle agitation for 8 weeks. Samples of the supernatant were collected at varying time intervals, and the amount of released PDGF-BB in solution was quantified by ELISA. The release was calculated and expressed as the ng amount of PDGF-BB at a given time point.

**Figure 5:**
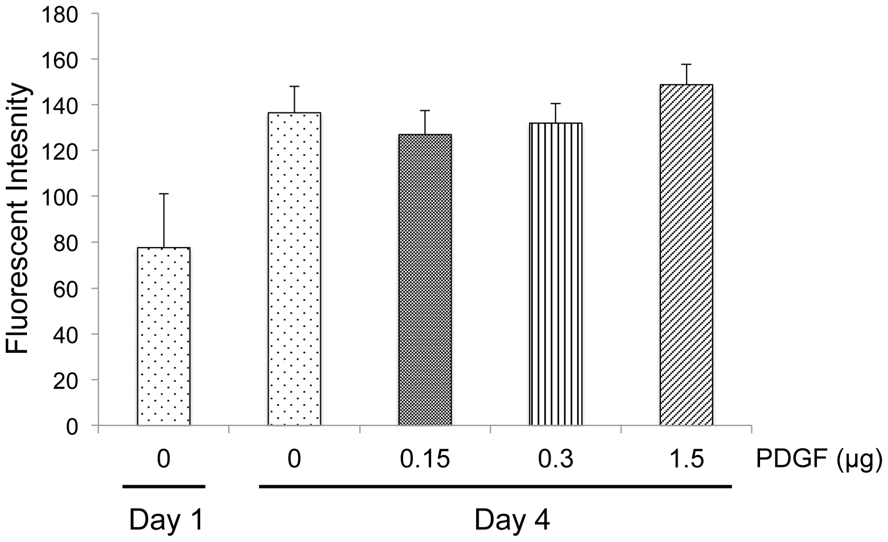
Mitogenic effects of PDGF-BB. GFP-MSCs (4×10^3^) were seeded onto PCL/col/HA scaffolds and grown in reduced serum media. Cell number was measured at one day on PCL/col/HA scaffolds and four days for scaffolds pre-coated with varying concentrations of PDGF-BB. MSCs were lysed and solution fluorescence of the released GFP was measured.

**Figure 6:**
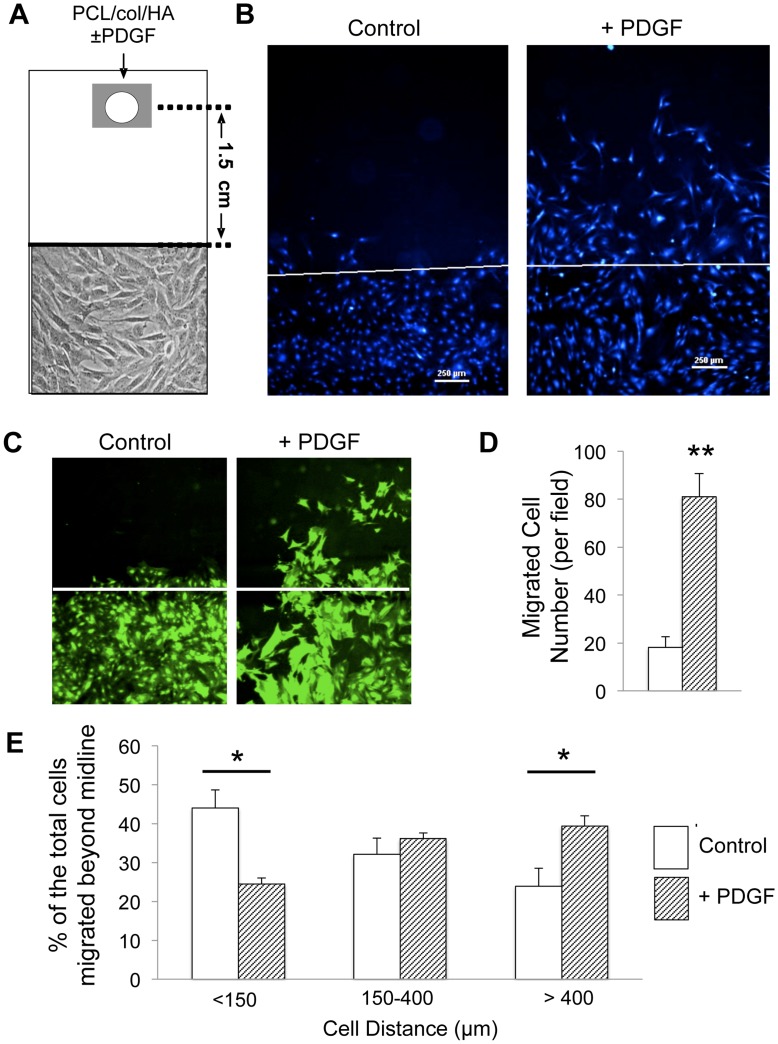
Released PDGF-BB induces chemotaxis of MSCs in a stringent migration assay. *A)* Schematic showing experimental set-up (not drawn to scale). GFP-MSCs were seeded in 8-well rectangular plates. After cell confluence was established, cells were completely removed from the top half of the well by scraping along a pre-drawn central line. Subsequently, a PDGF-BB-adsorbed PCL/col/HA/scaffold, placed on a steel wire mesh, was placed 1.5 cm away from the cell front. As a control, some chambers were set up with PCL/col/HA scaffolds lacking PDGF-BB. ***B)*** After a 72 hr-incubation in the chambers described, MSCs were stained with DAPI and visualized by fluorescence microscopy. The original cell front created is denoted by a white line. ***C)*** GFP-images showing change in cell morphology of MSCs exposed to PDGF-BB. ***D)*** Significantly greater cell number was observed migrating toward PDGF-BB coated scaffolds compared to uncoated scaffolds. ***E)*** DAPI-stained images were further analyzed by counting the number of cells in three defined regions of distance beyond the original cell front. The distribution of cells in the wells with PDGF-BB-coated scaffolds showed that a greater percentage of the total cells that had migrated beyond the cell front had localized to the region beyond 400 µm. In comparison, the greatest percentage of cells in the control wells localized to the region below 150 µm. A total of six samples were analyzed for each condition. An asterisk (*) denotes significant differences observed with *p*<.01, whereas (**) denotes *p*<.0001.

### Boyden Chamber Migration Assays

Chemotaxis of human GFP-MSCs was performed in Boyden chamber units with transwell inserts (Corning Inc. Corning, NY), 6.5 mm in diameter with 8 µm pore size filters. To facilitate initial cell attachment, the upper side of the insert filter was pre-coated for 2 h at 37°C with the following solution: α minimum essential medium (αMEM, Invitrogen, Grand Island, NY) containing 1% fetal bovine serum (FBS) and 0.25% (w/v) bovine serum albumin (BSA). Serum-free αMEM with 0.25% BSA (hereafter designated as “assay media”) was placed into the lower wells of the Boyden chambers. For some trials, chemotactic factors were added to the assay media in the lower chambers; these included PDGF-AB, and PDGF–BB, BMP-2, and a chemokine cocktail containing SDF-1α CXCL16, MIP-1α, MIP-1β, and RANTES (R&D Systems, Minneapolis, MN). After setting up the chambers with or without chemotactic factors, GFP-MSCs (4×10^4^) were seeded into the upper chambers and allowed to migrate at 37°C for 20 h. The cells on the upper face of the filter (non-migrating cells) were then removed by wiping 3 times with a wet cotton wool swab. Transmigration of GFP-MSCs to the bottom surface of the filter was visualized by a fluorescent stereomicroscope Leica MZ16F (Leica Microsystems, Bullerlo Grove, IL). Quantification of migrated cells was performed by trypsinizing cells on the underside, and lysing cells in 1% Triton X-100 in 50 mM Tris-HCl buffer, pH 7.5 (“lysis buffer”). Fluorescence of the lysates, due to released GFP, was measured by fluorometry. Data shown in Figure 1A and B are representative of three independent runs, each performed in duplicate.

In addition to experiments performed with purified recombinant chemotactic factors, we monitored chemotaxis toward PDGF-BB that had been released from scaffolds. Specifically, the conditioned media was collected from PDGF-BB-coated scaffolds that had been incubated in serum-free αMEM for 72 hr at 37°C with agitation. This solution was then placed in the lower chamber of a Boyden chamber, and migration of GFP-MSCs toward the PDGF-BB-containing conditioned media was monitored by measuring the fluorescence of cell lysates as described above. We also determined the concentration of released PDGF-BB within the media by ELISA. Purified PDGF-BB (10 ng/ml) and serum-free media were used as positive and negative controls, respectively. Three independent runs were performed in duplicate. Analysis of variance was carried out with StatPlus:mac LE (AnalystSoft Inc., www.analystsoft.com) with Tukey’s HSD post-hoc tests used to make pair-wise comparisons between groups. A confidence level of at least 95% (*p*<.05) was considered significant.

To validate the use of cell lysate fluorescence as a reporter for relative cell number, a standard curve was generated. GFP-MSCs were detached from tissue culture flasks by trypsinization and then counted with a hemocytometer. A defined number of cells was placed into an eppendorf tube and centrifuged. The supernatant was removed and the cell pellet resuspended in lysis buffer for 15 minutes at room temperature. Solution fluorescence of the released GFP was measured by fluorometry (Fig 1). Seven independent experiments were performed in duplicate with cells from passages 2–7. Linear regression and the coefficient of determination were determined using StatPlus:mac LE.

### GFP-MSC Proliferation on PDGF-BB Coated PCL/col/HA Scaffolds

PCL/col/HA scaffolds were coated with either 0, 0.15, 0.3, or 1.5 µg of PDGF-BB in 300 µL of PBS at 4°C for 24 h. Scaffolds were washed in PBS prior to being seeded with 4×10^3^ GFP-MSCs in 500 µL of reduced serum media (5% FBS). After 1 or 4 days of culture, cells were trypsinized from the scaffolds and then lysed in 1% Triton X-100 in 50 mM Tris-HCl buffer, pH 7.5. Solution fluorescence of the released GFP was measured by fluorometry. Three independent experiments were performed in duplicate.

### Modified MSC Migration Assay

GFP-MSCs were seeded at 8×10^4^ cells/well in 8-well rectangular tissue culture plates (Nunclon/Fisher Scientific,Pittsburg, PA ), and allowed to establish confluence. A central line was then drawn horizontally across the wells (on the underside of the dish), and the cells in the upper half of each well (above the line) were removed using a cell scraper. After removal of the cells, a scaffold was placed into the cell free side of the well at a distance 1.5 cm from the cell front created by the cell scraper. Specifically, scaffolds were pre-coated with or without 1.5 µg PDGF-BB for 24 hours, and then one scaffold per well was placed on a steel wire grid to suspend the scaffold in the media. The cultures containing scaffolds and GFP-MSCs were incubated at 37°C without further disturbance, which allowed the adsorbed PDGF-BB to release and diffuse through the media towards the cell front and stimulate migration. After 72 h of culture, cells were fixed, stained with DAPI, and migration was examined and imaged microscopically by using a fluorescent stereomicroscope Leica MZ16F.

To quantify cell migration, a total of 24 images were analyzed per sample (3 independent experiments performed in duplicate for a total of 6 wells per sample, 4 images per well). A custom MATLAB script was created to quantify the number and location of cells migrated across the cell front towards the scaffold. The script calculated the location of each cell nuclei that had migrated across the line created originally when manually removing cells. Cell number was analyzed using an unpaired Student’s t-test parametric analysis and analysis of variance for cell migration distances was carried out with StatPlus:mac LE with Tukey’s HSD post-hoc tests used to make pair-wise comparisons between groups. A confidence level of at least 95% (*p*<.05) was considered significant.

## Results and Discussion

### PDGF-BB is a Potent Chemotactic Factor for MSCs

The migration capacity of MSCs is influenced by a large range of growth factors, cytokines, and chemokines [8], [36], [37]. As a first step toward identifying an optimal chemotactic agent for delivery on bone-mimetic scaffolds, we used Boyden chamber assays to compare MSC migration in response to PDGF-AB, PDGF-BB, BMP2, or a chemokine mixture containing SDF-1α, CXCL16, MIP-1α, MIP-1β and RANTES. Chemoattractants, or serum-free media as a negative control, were added to the lower wells of transwell chambers, and GFP-expressing human MSCs were seeded in the upper chamber. After a 20-hr incubation, migrated cells on the underside of the filter were lysed and solution fluorescence was quantified. As shown in Fig 2A, the two PDGF isoforms, PDGF-AB and PDGF-BB, induced markedly greater chemotaxis than the chemokine mixture, and PDGF-BB also induced considerably more chemotaxis compared to BMP-2 (Fig 2B). Additionally, MSC response to PDGF-BB was observed in a dose-dependent manner with 10 ng/ml having the greatest effect (Fig 2C). These results are consistent with a growing literature suggesting that PDGF-BB is more effective than BMP-2 or CC/CXC chemokines in stimulating chemotactic activity of human bone marrow-derived MSCs. For example, Ozaki et al. tested 26 different growth factors/chemokines, and of these PDGF-BB had the greatest effect on MSC chemotaxis in multiple assays. Additionally, anti-PDGF-BB antibodies were able to inhibit PDGF-BB induced MSC migration [8]. RANTES, MIP-1α and MIP-1β (CC family) or SDF-1α and CXCL16 (CXC family) are chemokines involved in recruitment of immune cells to areas of inflammation and their receptors have also been shown to be expressed by human MSCs [37]. In our study, MSCs exhibited a low level of migratory response to the cocktail of the chemokines, although the response was dose-dependent. Given this weak response, the individual chemotactic profile of each chemokine in the cocktail was not further tested. Our results are in agreement with the findings of Ponte et al., who observed limited MSC chemotaxis toward RANTES, SDF-1, or macrophage-derived chemokine (MDC) [36]. Intriguingly, in this same study pre-treatment of MSCs with TNFα significantly enhanced MSC migration toward RANTES and SDF-1, which prompted these authors to suggest that these chemokines may play important roles in MSC homing to inflamed tissue sites. The osteoinductive protein, BMP-2, has also been suggested to serve as a chemotactic factor for selected cell types, including bone-associated cells. For instance, BMP-2 stimulates chemotaxis of human osteoblasts, bone-marrow derived osteoblasts, and human osteosarcoma cell lines [38]. However, in the current study BMP2 failed to elicit chemotaxis of MSCs. This may be due to phenotypic differences between MSCs and more differentiated osteoblastic cell types, or other variables relating to isolation or propagation of distinct cell cultures.

### Bone-mimetic Scaffolds Adsorb Greater Amounts of PDGF-BB as Compared with PCL Scaffolds

Prior studies from our group indicated that the inclusion of collagen I and nanoHA in electrospun PCL scaffolds increased the capacity of scaffolds to adsorb the adhesive proteins, fibronectin and vitronectin [34]. To evaluate the propensity of the bone-mimetic scaffolds to adsorb PDGF-BB, PCL/col/HA or PCL scaffolds were coated with solution containing 1.5 µg of PDGF-BB. We found that 1.37 µg ±0.02 and 0.83 µg ±0.08 of the protein were adsorbed to PCL/col/HA and PCL scaffolds, respectively, representing 91% and 55% of the total PDGF-BB in the starting solutions (Fig 3A). These results confirm that bone-mimetic scaffolds have an increased capacity for adsorbing PDGF-BB, relative to scaffolds composed of PCL alone.

### PCL/col/HA Scaffolds Release Greater Amounts of PDGF-BB over an 8-week Time Interval

To evaluate release kinetics, PCL/col/HA or PCL scaffolds were coated with PDGF-BB, washed briefly, and then resuspended in PBS. At varying time points, samples of the conditioned PBS were collected and monitored for PDGF-BB release using an ELISA assay (Fig 3B). It was found that a rapid release of PDGF-BB occurred within the first 4 days from the scaffolds, with greater amounts released from PCL/col/HA scaffolds, consistent with the greater loading capacity of this material. Continuous release was observed over an 8 week interval, with levels declining gradually. At every time point, a greater amount of PDGF-BB was released from PCL/col/HA, as compared with PCL scaffolds. These data suggest that PCL/col/HA scaffolds are suitable carriers for PDGF-BB.

### PDGF-BB Released from Scaffolds Stimulates MSC Chemotaxis

The adsorption of proteins onto biomaterial carriers can influence protein activity [39]; for example, many studies have shown that proteins can become denatured, or adopt altered conformations, when adsorbed to certain material surfaces. Thus it was important to test whether the PDGF-BB released from electrospun scaffolds was active. To this end, PCL/col/HA scaffolds were coated with PDGF-BB, washed briefly and then resuspended in serum-free media. After a 72-hr incubation, the conditioned media was collected and placed in the lower well of a transwell chamber. GFP-labelled MSCs were seeded into the upper chamber and allowed to migrate for 20 hrs. In addition, MSC migration was monitored in transwell chambers containing serum-free media with 10 ng/ml purified PDGF-BB (positive control) or serum-free media alone (negative control). As shown in Fig 4, PDGF-BB released from scaffolds induced a significant level of MSC migration compared to serum-free media, and no significant difference in MSC migration was observed between released and purified PDGF-BB (10 ng/ml). ELISA assays of the PDGF-BB released from scaffolds revealed a concentration of 12.465±3.557 ng/ml in the conditioned media used for Boyden chamber assays. These combined results show that the amount of PDGF-BB released from bone-mimetic scaffolds is sufficient to promote MSC migration, and also that adsorption to the scaffolds, and subsequent release, does not inhibit the bioactivity of the PDGF-BB.

### Mitogenic Effects of PDGF-BB

In addition to its chemotactic function, PDGF-BB is a known mitogen for many cell types including MSCs [40], [41]. Thus, we investigated whether MSCs would exhibit greater proliferation when adherent to PDGF-BB-coated scaffolds. PCL/col/HA scaffolds were pre-coated with varying concentrations of PDGF-BB and then GFP-MSCs were seeded onto the scaffolds and incubated for four days. Relative cell number was quantified by lysing cells and measuring fluorescence. As seen in Fig 5, no significant difference was observed between the uncoated and PDGF-BB-coated samples. It is possible that this lack of effect may be due to the presence of suboptimal PDGF-BB concentrations for inducing mitosis. Alternatively, PDGF-BB may not be able to stimulate MSC proliferation beyond the level stimulated by PCL/col/HA scaffolds themselves, as we have previously shown that PCL/col/HA scaffolds promote significantly greater MSC proliferation than PCL scaffolds. While further studies will be needed to determine the reason for the lack of mitogenic activity of PDGF-BB when coupled to scaffolds, these results suggest that under the conditions used in this study, the principal benefit of adsorbed PDGF-BB is in its function as a chemotactic, rather than proliferative, factor.

### PDGF-BB Released from Scaffolds Stimulates MSC Chemotaxis in a Stringent Migration Assay

Boyden chamber assays represent a standard method for evaluating chemotaxis, however *in vivo*, chemotactic gradients may need to act over greater distances, and rapid dilution of the factors can occur. Accordingly, we developed a more stringent chemotaxis assay to better model the capacity of PDGF-BB-modified bone-mimetic scaffolds to influence endogenous MSC recruitment. As diagrammed in Fig 6A, MSCs were grown to confluence in rectangular tissue culture wells; a defined region of the cell cultures was then removed using a cell-scraper to create a distinct cell front. A PDGF-BB-coated PCL/col/HA scaffold, suspended on a steel wire grid, was placed at one end of the well, at a distance of 1.5 cm from the cell front. As controls, cell cultures were set up with a PCL/col/HA scaffold lacking PDGF-BB. After a 72 h incubation, DAPI staining of cell nuclei and subsequent fluorescent imaging revealed robust MSC migration toward the PCL/col/HA scaffolds coated with PDGF-BB, but very little toward uncoated PCL/col/HA scaffolds, indicating that PDGF-BB released from scaffolds was effective in stimulating chemotaxis even when the source of PDGF-BB was 1.5 cm from the cell front (Fig 6B). In addition, imaging of GFP-labeled MSCs showed that cells in wells with PDGF-BB-coated scaffolds exhibited an altered morphology (Fig 6C), consistent with the known effects of PDGF on cell shape [9], [42], [43]. In order to quantify the number and location of cells migrating beyond the original cell front, a custom MATLAB script was created to decrease processing time and remove user bias. The script used an algorithm to locate clusters of connected pixels to identify DAPI-stained cell nuclei, and then calculated the distance from the center of the nuclei to the original cell front. Results from this analysis showed that wells with PDGF-BB-coated scaffolds had a significant increase (p<.0001) in the total number of MSCs migrating beyond the original cell front (Fig 6D). As an additional measure of chemotaxis, we subdivided the area above the cell front into defined regions, and calculated the percentage of cells within each of these regions relative to the total number of cells that had crossed the central line. These data showed that cells exposed to PDGF-BB-releasing scaffolds migrated significantly greater distances than cells incubated with uncoated bone-mimetic scaffolds (Fig 6E). Finally, it is noteworthy that the tissue culture wells that the scaffolds were placed into contained 4.5 mL of media, resulting in a substantial dilution of the released PDGF-BB. Thus, PDGF-BB released from PCL/col/HA scaffolds can induce MSC migration under conditions of greater dilution and distance than typically employed in standard Boyden chamber assays.

### Conclusion

In the present study we show that bone-mimetic electrospun scaffolds composed of PCL, collagen I and nanoparticulate HA have a greater capacity to adsorb and release PDGF-BB than scaffolds composed of PCL alone, and release is sustained for at least 8 weeks. Furthermore, the PDGF-BB released from the PCL/col/HA scaffolds is effective in stimulating chemotactic migration of MSCs under stringent assay conditions. These collective results suggest that electrospun scaffolds incorporating the bone matrix molecules, collagen I and HA, not only provide favorable matrices for MSC attachment and proliferation, but also serve to concentrate and deliver growth/chemotactic factors, much like native extracellular matrices. In sum, PDGF-BB-modified bone-mimetic scaffolds represent promising materials for bone regenerative therapies.
